# LEF1‐AS1, long non‐coding RNA, inhibits proliferation in myeloid malignancy

**DOI:** 10.1111/jcmm.14152

**Published:** 2019-02-15

**Authors:** Ada Congrains-Castillo, Fernanda S. Niemann, Adriana S. Santos Duarte, Sara T. Olalla‐Saad

**Affiliations:** ^1^ Hematology and Hemotherapy Center, Hemocentro­Unicamp São Paulo Brazil

**Keywords:** LEF1-AS1, long non-coding RNA, acute myeloid malignancy

## Abstract

LEF1 antisense RNA 1 (LEF1‐AS1) is an antisense long non‐coding RNA encoded in the lymphoid enhancer‐binding factor 1 (LEF1) locus. LEF1‐AS1 is a conserved transcript dysregulated in hematopoiesis. This study aimed to functionally characterize the role of this transcript in myeloid malignancy and explore a possible regulatory effect of LEF1‐AS1 upon LEF1. We show that LEF1‐AS1 is highly expressed in normal hematopoietic stem cells but barely detectable in myeloid malignant cell lines. Additionally, bone marrow cells from myelodysplastic syndrome (n=12) and acute myeloid malignancy patients (n=28) expressed significantly reduced levels of LEF1‐AS1 compared to healthy controls (n=15). Artificial LEF1‐AS1 over‐expression inhibited proliferation in HL60 and led to an upregulation of tumor suppressors p21 and p27, and reduced ERK1/2 activation. Unexpectedly, no underlying modulation of LEF1 was detected. Ectopic expression of LEF1‐AS1 also inhibited proliferation in HELA, a cell line lacking endogenous expression of LEF1, supporting a LEF1‐independent mechanism. Additionally, transient over‐expression of LEF1‐AS1 in AML patient cells also led to reduced proliferation and colony formation capacity. We used a mass spectrometry‐based proteomics approach. Proteomic quantification identified the modulation of an important metabolic regulator, Fumarase, and concomitant accumulation of the metabolite fumarate.

Acute myeloid leukaemia (AML) is an aggressive haematologic disorder and despite advances in diagnosis and treatment, AML‐related mortality remains high. Acute myeloid leukaemia is characterized by accumulation of undifferentiated cells in the bone marrow and blood. Aberrant control of cell growth and metabolism in haematopoietic precursors appear as underlying mechanisms of leukaemogenesis. Unraveling the molecular mechanisms that control proliferation in myeloid cells is crucial for the development of new therapeutic approaches. In this sense, long non‐coding RNAs (lncRNAs) have emerged as major players in disease pathogenesis.^1^, ^2^

Lymphoid enhancer‐binding factor 1 (LEF1) Antisense RNA 1, LEF1 antisense RNA 1 (LEF1‐AS1), is a highly conserved transcript and several lines of evidence suggest an important role of this lncRNA in the haematopoietic system.^3^, ^4^, ^5^ However, this is the first study, to our knowledge, to characterize the role of LEF1‐AS1 in myeloid cells.

A recent study identified an unprocessed non‐coding transcript in the locus and demonstrated that this transcript regulates LEF1 coding expression in pancreatic and colorectal carcinoma cell lines.^6^ The function of LEF1‐AS1 in these cell lines was dependent on the unspliced transcript and ultimately on the regulation of LEF1.^6^ Based on this evidence and as LEF1 coding gene has an established role in myeloid malignancy,^7^, ^8^ we suspected a cis‐regulatory mechanism between the antisense non‐coding transcript and LEF1 in the haematopoietic system. To investigate the role of LEF1‐AS1 in the regulation of LEF1 and myeloid malignancy pathogenesis, we used a stable transfection approach to overexpress the transcript in myeloid cell line HL60. Stably transfected cells were obtained by DMRIE(1,2‐dimyristyloxypropyl‐3‐dimethyl‐hydroxy ethyl ammonium bromide) ‐C‐mediated transfection using pcDNA vector containing full length LEF1‐AS1 or empty pcDNA vector, and 3 weeks of geneticin selection. This method was adapted from Grinstein et al^9^ and details are presented in Supplementary information. Unexpectedly, we could not detect any significant alteration of LEF1 expression after overexpression of LEF1‐AS1 (Figure 1A). Despite the lack of effect upon LEF1, we observed that LEF1‐AS1 overexpression led to inhibition of proliferation as shown by two different proliferation‐specific assays. Carboxyfluorescein succinimidyl ester (CFSE) labelling was used to trace cell divisions in HL60 stably transfected cells. Flow cytometry analysis showed that cells overexpressing LEF1‐AS1 underwent less cell divisions (Figure 1C; Supplementary). Additionally, flow cytometry measurement of Ki67 staining (proliferation marker) was also was significantly reduced in LEF1‐AS1‐HL60 synchronized cells (Figure 1D).

**Figure 1:**
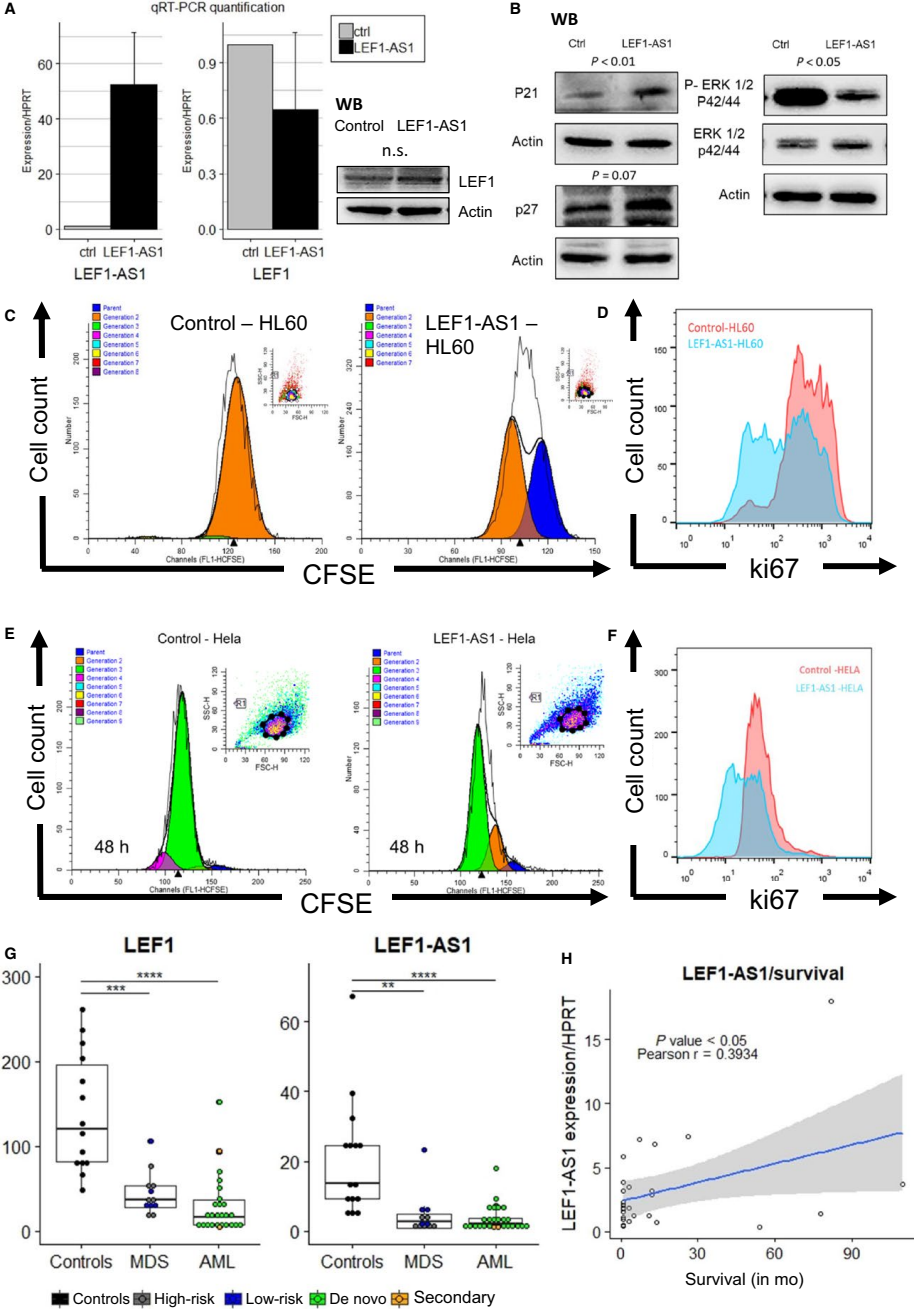
A, Relative quantification of LEF1 gene shows no significant modulation of LEF1 at mRNA level in HL60 and efficient overexpression of LEF1‐AS1, immunoblot of LEF1 protein in cell lysates of controls and LEF1‐AS1 overexpressing HL60 cells (n = 3, see Supplementary results). B, Immunoblots for anti‐P21, anti‐p27, anti‐ERK1/2 and anti‐phosphoERK1/2 of control and LEF1‐AS1 cells (see results from independent experiments in Supplementary), statistical analysis was performed using the relative optical density of the bands (Alliance software, Uvitec Ltd.). C, Carboxyfluorescein succinimidyl ester (CFSE) cell division tracking assay of HL60 overexpressing empty vector and LEF1‐AS1. Carboxyfluorescein succinimidyl ester fluorescence was measured by flow cytometry. Blue peak: parent cells, orange peak: first generation (n = 3). D, HL60 cells were labelled with allophycocyanin‐conjugated ki67 antibody and analysed by flow cytometry, LEF1‐AS1‐HL60 showed less positive Ki67 cells and a reduced overall intensity of the proliferation marker Ki67 (n = 3). E, Carboxyfluorescein succinimidyl ester cell division tracking assay of empty vector and LEF1‐AS1‐Hela. Carboxyfluorescein succinimidyl ester fluorescence was measured by flow cytometry. Blue peak: parent cells, orange peak: first generation, green peak: second generation, purple peak: third generation. (n = 3, see Supplementary). F, LEF1‐AS1‐Hela showed less positive Ki67 cells and a reduced overall intensity of the proliferation marker Ki67(SI) (G) qRT‐PCR quantification of long non‐coding transcript, LEF1‐AS1 and LEF1 in normal bone marrow cells of 15 controls, 12 myelodysplastic syndrome patients (MDS) and 28 acute myeloid leukaemia patients bone marrows (AML). Lower relative expression levels were detected in patients compared with controls. H, Correlation and linear regression between expression of LEF1‐AS1 and survival time after diagnosis

In line with this reduction in proliferation, an increased expression of tumour suppressors CDKN1A (p21) and CDKN1B (p27) was detected in the mRNA and protein levels (Figure 1B; Supplementary). A reduction of ERK1/2 activation was also detected by western blot, without modulation of ERK1/2 expression (Figure 1; Supplementary). We observed no difference in apoptosis levels as shown by annexin‐V cytometric analysis (Supplementary). To evaluate definitely, if the phenotypical effects of LEF1‐AS1 overexpression were mediated by LEF1 function, we also overexpressed LEF1‐AS1 in Hela (methods in Supplementary), a cell line lacking endogenous expression of LEF1 . We observed a reduction in proliferation in LEF1‐AS1 overexpressing cells using CFSE‐mediated cell division tracking and Ki67 staining (Figure 1E,F), and no effect upon LEF1 expression (no detection by RT‐PCR in control and LEF1‐AS1 cells).

Lacking an obvious candidate mediator of this anti‐proliferative function of LEF1‐AS1, we resorted to a mass spectrometry‐based proteomics approach to characterize the function of LEF1‐AS1. Whole‐cell lysates were processed and analysed by Q‐tof mass spectrometry. A total of over 500 proteins were identified (see complete list in a supplementary file) and 16 were differentially expressed (*P*‐value cut‐off of 0.05, see Table S2). We were able to validate the most relevant results by western blot (Figure 2A). Histone 3 (H3), a marker of proliferation,^10^, ^11^ was reduced, in line with our functional results. Talin is a protein involved in cell‐to‐cell and cell‐to‐substrate adhesion and migration and it has a role in metastasis of several cancers, but its contribution to leukaemogenesis is not clear. Talin up‐regulation was also observed in Hela and may play a role in LEF1‐AS1 function in other tissues (Supplementary). We also validated the increased protein expression of RAB7A, a small GTPase that regulates exocytosis/endocytosis‐mediated protein/RNA trafficking.^12^ Remarkably, the activation of RAB7A is associated with the increased endocytic degradation of epidermal growth factor receptor,^13^ which suggests a protective role of this protein against leukaemogenesis. Among the modulated proteins, fumarase or fumarate hydratase (FH) drew our attention as potential mediator of the observed reduction in proliferation (Figure 2A). The down‐regulation of fumarase is accompanied by the consequent intracellular accumulation of fumarate (Figure 2B). Remarkably, FH inhibition reduces proliferation in THP1, a myeloid cell line,^14^ and inhibition of FH in haematopoietic cells prevents leukaemic transformation, suggesting it may be a player in the anti‐proliferative effect of LEF1‐AS1 observed in our experiments.

**Figure 2:**
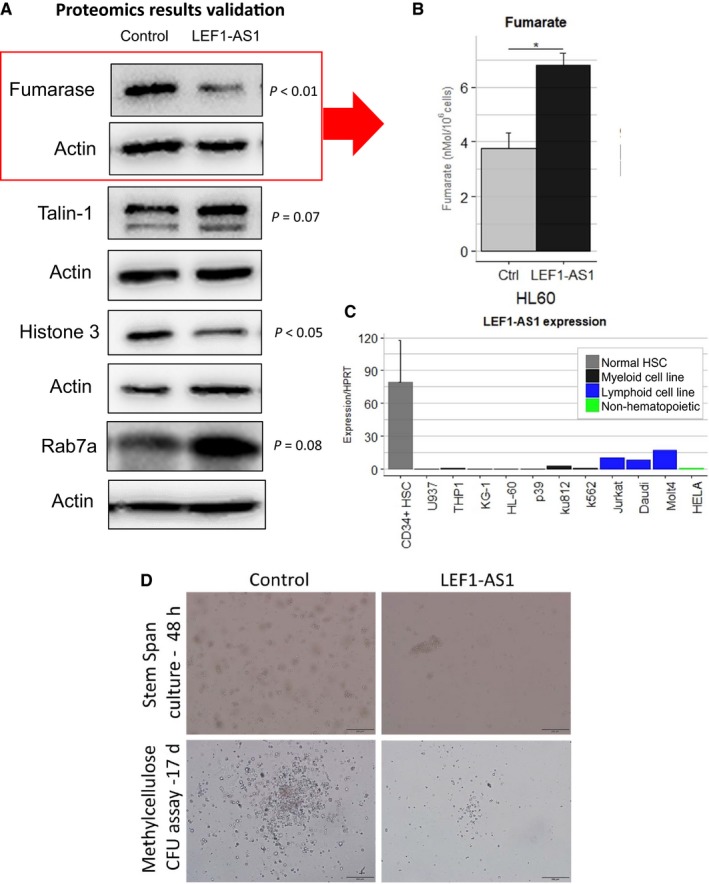
A, Validation of proteins modulated by LEF1‐AS1 detected by mass spectrometry using western blot, statistical analysis (paired *t* test) was performed using the relative optical densities of the bands of three independent experiments (see bands used in the analysis in Supplementary). B, Measurement of fumarate levels between control and LEF1‐AS1‐HL60 cells. C, Relative expression of LEF1‐AS1 in CD34+haematopoietic stem cells (from umbilical cord donors, n = 3), several myeloid, lymphoid cell lines and Hela (n = 1 of each). D, Microscopic images of bone marrow mononuclear cells of AML patient after 48 h in expansion medium (upper panels) and representative colonies after 17 d in methylcellulose culture (lower panels). Cells nucleofected with control (left panels) empty vector and LEF1‐AS1(right panels) containing vector

LEF1‐AS1 and LEF1 expressions in myeloid malignancy were quantitatively evaluated by quantitative real‐time polymerase chain reaction (qRT‐PCR) using mononuclear cells isolated by Ficoll‐Hypaque separation from bone marrow samples of 15 controls, 12 MDS patients and 28 AML patients. We observed a dramatic reduction of LEF1‐AS1 expression in MDS and AML patients when compared to healthy bone marrow donors (controls = 15, MDS patients = 12, fivefold reduction, *P* = 0.0042, AML patients = 28, sevenfold reduction, *P* < 0.0001) (Figure 1G). The expression of LEF1 was also reduced (controls = 14, MDS patients = 12, *P* = 0.0002, AML patients = 27, *P *< 0.0001) (Figure 1G). Although, there is a significant age difference between the groups, no significant correlation was found between LEF1 or LEF1‐AS1 expression and potential confounders such as age and percentage of blasts in the bone marrow (results section of Supplementary). The suppression of LEF1 in myelodysplastic syndrome is well documented^8^; however, the expression pattern of LEF1 in AML had been less clear, and high expression of LEF1 has been associated with a favourable prognosis in a subtype of AML.^15^ Here, we show that LEF1 is suppressed in a heterogenous sample of AML and we could not correlate expression of LEF1 with patient outcome. Interestingly, despite strong correlation between LEF1 and LEF1‐AS1 expression (Supplementary), only LEF1‐AS1 expression was positively correlated with AML patient survival (LEF1‐AS1: *P* value (two‐tailed) = 0.0423 Pearson r = 0.3934, 95% confidence interval = 0.01567‐0.6729), Figure 1H. Supporting these results, normal haematopoietic stem cells (CD34+HSCs) express high levels of LEF1‐AS1 when compared to malignant cell lines and LEF1‐AS1 expression is particularly suppressed in myeloid malignant cells (Figure 2C).

We next examined the anti‐proliferative effects of LEF1‐AS1 in mononuclear cells from an AML patient. Transient overexpression of LEF1‐AS1 in these cells using Amaxa nucleofector caused a dramatic reduction in their colony formation capacity (17‐days methylcellulose CFU assay) and a clear reduced cell number after 48 hours of culture in expansion medium Stem Span, when compared to empty‐vector nucleofected cells (Figure 2.D). Methylcellulose colony‐forming unit (CFU) assay showed that the number of cells capable of forming leukaemic cell colonies was reduced as well as colony size in LEF1‐AS1 nucleofected cells after 17 days in semi‐solid culture (control: 54 colonies, LEF1‐AS1: 14 colonies), see Figure 2D and details in Supplementary information. RNA was isolated 48 hours after nucleofection, showing efficient overexpression of LEF1‐AS1 and no effect upon LEF1 coding gene (Supplementary).

We observed that LEF1‐AS1 is lost in myeloid malignant cells. Expression of LEF1‐AS1 was shown to be reduced in haematopoietic stem cells from myelodysplastic syndrome patients,^3^ we observed the same pattern in total bone marrow cells. MDS is a haematologic disorder characterized by blood cytopenia and increased risk of developing AML.^3^ This loss of expression is also observed in AML patients’ bone marrows suggesting this suppression may be an important step in malignization and disease progression. As we demonstrated, the artificial re‐expression of LEF1‐AS1 reduces proliferation of myeloid cell line HL60, non‐haematopoietic Hela and AML patient mononuclear cells. Although, the mechanism by which LEF1‐AS1 regulates cell proliferation remains unclear, our results strongly suggest that LEF1‐AS1 has a protective anti‐proliferative role in myeloid malignancy and future work is required to understand the molecular functions and implications of this transcript in other pathologies.

## CONFLICT OF INTEREST

The authors confirm that there are no conflicts of interest.
