# Supplementary material for: LEF1‐AS1, long non‐coding RNA, inhibits proliferation in myeloid malignancy
Source: J Cell Mol Med. 2019 Feb 15;23(4):3021–5. doi: 10.1111/jcmm.14152 (PMC6433713; doi:10.1111/jcmm.14152)

**Supplementary material**

LEF1-AS1, long non-coding RNA, inhibits proliferation in myeloid malignancy

A. Congrains, F. Niemann, F. Corrocher, S.T. Olalla-Saad

Hematology and Hemotherapy Center, Hemocentro­Unicamp, Campinas, São Paulo, Brazil

Corresponding author: Ada Congrains, email: [adacongrains@hotmail.com](mailto:adacongrains@hotmail.com), mailing address: Rua Carlos Chagas 480, Barao Geraldo, Campinas, SP, Brazil/Unicamp, phone number : +55(19) 32895438

**Supplementary Materials and Methods**

**Patients**

Samples from 28 patients with previously untreated AML and 12 patients with MDS by the World Health Organization (WHO) criteria, confirmed by cytologic examination of blood and bone marrow were used in this study. Mononuclear cells were isolated by Ficoll-Hypaque separation from total bone marrow (BM) samples from MDS patients (6 males, 6 females, median age 72.5, range 31-86) and AML patients (12 males, 16 females, median age, 59 years, range, 27-93 years) were collected at the time of diagnosis and BM mononuclear cells of 15 controls (12 males, 3 females, median age, 30 years, range, 15-47 years) were obtained from bone marrow donors. French - American - British (FAB) classification of the patients is presented in table 1. All patients were diagnosed between 2009 and 2014 at the hematology and hemotherapy center, University of Campinas hospital and gave written informed consent to the study, all procedures were approved by the University Ethics Committee.

**Supplementary table 1.** Patients characteristics

| **Patient Characteristics** | | |
| --- | --- | --- |
|  | Number | Relative LEF1-AS1 expression (Mean) |
| **Controls** | 15 | 20.53 |
| Sex (Male/female) | 12/3 |  |
| Age median [range] | 30 [15-47] |  |
| **MDS cases** | 12 | 4.50 |
| Sex (Male/female) | 6/6 |  |
| Age median [range] | 72.5 [31-86] |  |
| % of blasts in BM, mean | 5.4% |  |
| FAB classification |  |  |
| Low Risk (RA/RARS) | 4/1 |  |
| High Risk (RAEB/RAEB-t) | 6/1 |  |
| **AML cases** | 28 | 3.40 |
| Sex (Male/female) | 12/16 |  |
| Age median [range] | 59[27-93] |  |
| % of blasts in BM, mean | 74.54% |  |
| FAB classification |  |  |
| M0 | 3 |  |
| M1 | 8 |  |
| M2 | 4 |  |
| M3 | 3 |  |
| M4 | 4 |  |
| M5 | 3 |  |
| AML-MRC (secondary to MDS) | 3 |  |

RA: Refractory Anemia

RARS: Refractory anemia with ringed sideroblasts

RAEB: Refractory Anemia with Excess Blasts

RAEB-t: Refractory anemia with excess blasts in transformation

M0: Undifferentiated acute myeloblastic leukemia

M1: Acute myeloblastic leukemia with minimal maturation

M2: Acute myeloblastic leukemia with maturation

M3: Acute promyelocytic leukemia

M4: Acute myelomonocytic leukemia

M5: Acute monocytic leukemia

AML-MRC : Acute myeloid leukemia with myelodysplasia-related changes

**Cell culture, stable transfection and nucleofection**

HL60 cells were maintained in RPMI 1640 medium supplemented with 10% fetal bovine serum, HELA cells in Dulbecco's modified Eagle's medium supplemented with 10% fetal bovine serum. Stably transfected cells were obtained by DMRIE-C mediated transfection using pcDNA vector containing full length LEF1-AS1 or empty pcDNA vector, adaptation of the protocol presented in Grinstein et al [16]. Selection was carried out by addition of 500 μg/ml and 800 μg/ml geneticin to the culture medium of HL60 and Hela transfected cells for 3 weeks or until cells grew at similar rate than untransfected cells.

Bone marrow mononuclear cells from the acute promyelocytic leukemia patient (AML-M3) (1x10^6^ per nucleofection) were resuspended in P3 Primary Cell 4D-Nucleofector™ X Kit buffer and nucleofected using Amaxa™ 4D-Nucleofector™. After nucleofection cells were maintained in Stem spam medium supplemented with 10ng/ml of thrombopoietin (TPO), stem cell factor (SCF) and FlT3-ligand (FL), interleukin 3 (IL-3) and IL-6 in culture until RNA collection or plated in Methocult® H4534 Classic without EPO for 17 days for Colony formation Unit (CFU) assay (2x10^4^ cells in 1 ml of methylcellulose medium in a 6-well plate).

**RNA extraction and reverse transcription**

RNA was isolated from cell lines and total bone marrow samples using Illustra RNAspin Mini kit (GeHealthcare Life Sciences) following the manufacturer's instructions. RNA quantification was performed in a NanoDrop spectrophotometer (ND-1000 Spectrophotometer). A total of 1 μg of RNA from each sample was reverse transcribed into cDNA (RevertAid First Strand cDNA Synthesis Kit, ThermoScientific) using dT oligos with exception of the samples for PCR amplifications of unspliced LEFNAT.

**Quantitative RT-PCR (qRT- PCR)**

Real-time PCR amplifications were performed on the ABI 7500 Sequence Detector System (Applied Biosystems) using SybrGreen PCR Master Mix (Applied Biosystems). Primer sequences are presented in supplementary table 1. PCR reactions were performed in triplicates and HPRT was used as endogenous control for normalization, with exception of the samples of exosome-RNA , GADPH was used for those samples. Relative quantification of each normalized gene expression by the endogenous control was calculated using the formula 2-ΔCT. For patients quantification, cDNA from THP1 was used as inter-run calibrator sample on each plate to normalize for plate-to-plate variation and the expression of THP1 was arbitrarily set as 1 in graphs (THP1 expression was comparable to AML bone marrow expression levels).

**Supplementary figure 1.** Transcriptional map of LEF1-AS1/LEF1 locus and localization of rt-PCR primers.

**
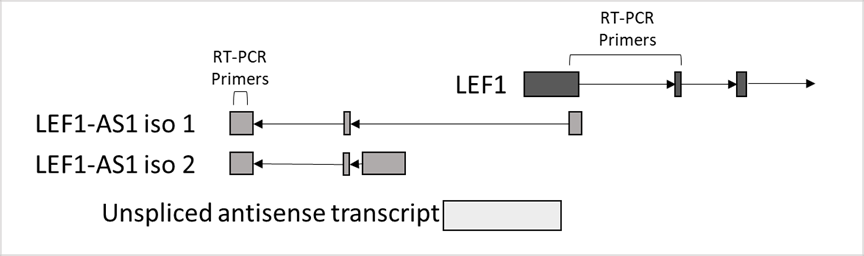
**

**Supplementary table 2. qRT-PCR primers list**

| Gene name | Primer forward | Primer reverse |
| --- | --- | --- |
| LEF1 | TTCCTTGGTGAACGAGTCTGAA | TCGTGGTAGGGCTCCTGAGA |
| LEF1-AS1 | GCATTGGGCAAGCACGTAA | ACCTTGTTCTGCATTAATCTCCCTAT |
| CDKN1A(p21) | TCACTGTCTTGTACCCTTGTGC | GGCGTTTGGAGTGGTAGAAA |
| CDKN1B(p27) | GAAGCCTGGCCTCAGAAGAC | CCATTCCATGAAGTCAGCGAT |
| ERK 1 | ACCTGCGACCTTAAGATTTGTGA | AGCCACATACTCCGTCAGGAA |
| ERK2 | TTTGTCAGGACAAGGGCTCA | TCAAAGGAGTCAAAGTGGATAAGC |
| HPRT (endogenous control) | GAACGTCTTGCTCGAGATGTGA | TCCAGCAGGTCAGCAAAGAAT |
|  |  |  |
|  |  |  |
|  |  |  |

**Supplementary table 3. Cloning primers**

| Gene name | Primer forward | Primer reverse |
| --- | --- | --- |
| LEF1-AS1 | CTCTCGGAACTGGGGCAG | GCTCACCTCGTGTCCGTT |
| NAT-unspliced LEF1 | CTCTCGGAACTGGGGCAG | CCCTTGTCTCCAAAGAGCG |

**Antibodies used for western blotting**

Cells were lysed in a buffer containing 100 mM Tris (pH 7.6), 1% Triton X-100, 150 mM NaCl, 0.1 mg Aprotinin, 35 mg /mL PMSF 10 mM Na3VO4, 100 mM NaF, 10 mM Na4P2O7, and 4 mM EDTA. Samples were centrifuged at 12000 RPM, 4 °C for 20 minutes to remove cell debris. Protein concentration was measured using the Bradford assay (Bio-Rad). Laemmli buffer containing 100 mmol/L of dithiothreitol was added to the protein extracts and heated at 100 °C for 5 minutes, Samples were run on 8%, 10% or 15% SDS-PAGEs. After the run, the proteins were transferred to nitrocellulose membranes (Millipore). Membranes were immunoblotted with specific antibodies:

Anti-P21 (abcam, ab7960), anti-p27 (Santa Cruz, sc-1641), anti-LEF1 (Cell signaling, #2286), anti-Talin1 (Santa Cruz, sc7534), anti-histone 3 (Santa Cruz, sc8654), anti-Fumarase (Abcam, ab171948), anti-ERK1/2 p44/42 (cell signaling, #9102S), anti-P-ERK1/2 P-p44/42 (T202/Y204)(cell signaling), anti-actin (Santa Cruz, sc1616).

**CFSE Cell proliferation**

Cells were counted, washed with PBS and resuspended in PBS containing 5% FBS. CFSE stock solution in DMSO according to manufacturer’s instructions and diluted in 5% FBS PBS and added to resuspended cells to a final concentration of 1 μM.

After 5 minutes incubation at 37 °C and 10 min on ice, cells were washed 3 times with PBS and then cultured in complete medium until fluorescence measurement in FACSCalibur and analysed using Modfit software.

**Ki67 staining**

Cells were synchronized in serum-free medium for 48 hours. For Ki67 analysis, 8 hours after release from starvation cells were fixed (as described above) and incubated with Ki67 APC-conjugated antibody in a solution of PBS, 1% FBS, 0,09% NaN3, pH7.2 for 30 minutes. After washing, 5 ul of propidium Iodide was added before measurement in FACSCalibur according to manufacturer`s instructions. Results were analyzed using FlowJo software.

**Protein digestion for mass spectrometry**

For protein digestion, 50 ug of protein from each sample was treated with a final concentration of 1.6 M urea, following reduction (5 mM dithiothreitol, 25 min at 56°C), alkylation (14 mM iodoacetamide, 30 min at room temperature in the dark) and digestion with trypsin (1:50, w/w). The reaction was stopped with 1% TFA and desalted with stage tips. The samples were dried in a vacuum concentrator, reconstituted in 0.1% formic acid and before LC-MS/MS analysis.

**Mass spectrometry**

An aliquot of 4.5 ul of tryptic peptides was were separated by C18 (100 mm6100 mm) RP-nanoUPLC (nanoAcquity, Waters) coupled with a Q-Tof Premier mass spectrometer (Waters) with nanoelectrospray source at a flow rate of 0.6 ml/min. The gradient was 2–90% acetonitrile in 0.1% formic acid over 45 min. The nanoelectrospray voltage was set to 3.5 kV, a cone voltage of 30 V and the source temperature was 100uC. The instrument was operated in the ‘top three’ mode, in which one MS spectrum is acquired followed by MS/MS of the top three most-intense peaks detected. After MS/MS fragmentation, the ion was placed on exclusion list for 60 s and for the analysis of endogenous cleavage peptides, a real time exclusion was used.

**Fumarate measurement**

Fumarate was measured using colorimetric assay, Fumarate assay kit (abcam ab102516). Control and LEF1-AS1 cells (1x106) were washed, homogenized and assayed according to manufacturer’s instructions. Absorbance was measured at 450 nm using a microplate reader, and concentrations were calculated based on the calibration curve provided in the kit.

**Statistical methods**

Statistical analysis of the data was performed using Graphpad prism software. The patient’s data was analyzed by Student’s t-test (two-way) and statistical significance between 2 groups (controls vs. MDS, and controls vs. AML) was shown in the graphs. The Pearson coefficient was used to measure correlations between LEF1 and LEF1-AS1 expression and LEF1-AS1/survival, Pearson r and P-value are shown in the corresponding figure. Gene ontology enrichment was carried out using String software V10.5, FDR (false discovery rate) P values were generated by String software and plotted using “R”. For the functional experiments the significance of differences between two groups (empty vector and LEF1-AS1) was estimated with Student's t test and differences were considered statistically significant at the level of P < 0.05. Optical densities for statistical analysis of WB bands were normalized by actin optical density (p-ERK band was normalized by ERK and OD of the control was arbitrarily set as 1), paired t-test was performed and P values are shown in the figures. All graphs were plotted using ‘R’ graphic packages.

**Results**

**LEF1-AS1 and LEFNAT Polymerase chain reaction amplification**

PCR for LEF1-AS1

LEF1-AS1, spliced transcript, as expected did not amplify from genomic DNA, but it showed a distinctive band in the expected size using several cell lines cDNA.

**Supplementary figure 2.** Gel stained with bromide ethidium, showing the products of amplification of the complete LEF1-AS1 transcript from several cell lines cDNAs.


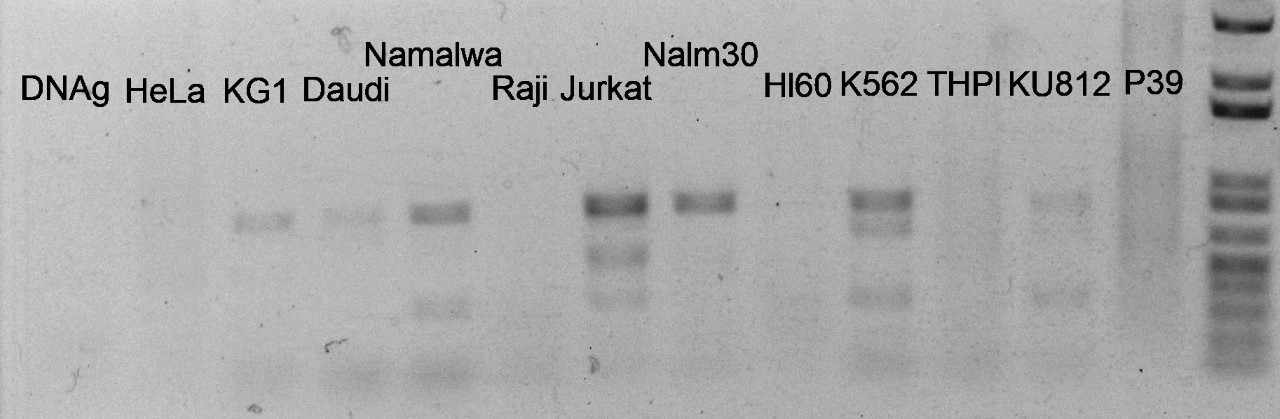


LEF1-AS1

~ 1 kb

Sequence obtained from the band of cDNA from Daudi cell line:

GCCGCCATGGCGGCCGCGGGAATTCGATTAAGGGGATCATCTCGTCCGTGGCGCAGAGTTCCGGGTCCCCCCCGCCGCCGCCACCTCCTCCGGAGAGTTGGGGCATCCCGGCGGCTCTGTAATCTCCGCTCCGCTGTGGGAGCACCCGCGCAACAGCAGGAAAGACAGAGGGGCCCCTTTGTGTGACTAAATTTGGCAAGAATATGGATCTGAGCCAGTCTTTCCACGTGTGCTCCCAGCTCCCAGCTGAGAAGGCCAAGGGTTCTCTTCCGGATGCAGCCTTTTTGAAATTTATTCTTGGCCAAAAGAGGAAGATAGTTCAAGGATTCTATCTTGCTTTTGGGGGGCAATGGAGAAACCCTGAAAACACTTAAGGAAAACCTGAGGCCTTCTATAAGAAAATAAACTCCCTGAAAGGGGAGGCAGGGCCAAGGAATTTACTTATTTTTATAAATCTCTTTCCTGGGTGGATATGGTAATGGATGTGTTTCTTGAATTGTGGTAAATGTACCTCATAAAATGGATGGACTGGGGCTATATTCTCAGGCTTCCTGGGCTCTGTTGGCATTGGGCAAGCACGTAATGCCTGGTTTGAGGCTAGAAGCCAAAGCCACAACCTTCTTGCAGTACCAACTGAGCCATAGGGAGATTAATGCAGAACAAGGTCGTTAGTCTCATTGTTTATCCAGTTACTAGCTGCATAGATTTTGTGTAAATTTCAGCTACAGTGGTTGCAGCCTGCCTTTGATGTGTGTAATCTGTACATTTTAGTGCTCCAGGAAAGTACCTGATTGACAAGATCTACATCCAATGCCCAGAAAACACATCCTACACAGGCACAGTGAAGCGTGAGTTGAAGAATTTTCCAGTGAGTTCCATCAAAGTAA

**PCR of LEFNAT (unspliced transcript)**

An interaction between the unspliced transcript LEF1NAT and LEF1-AS1 was previously reported in other cell models[10], therefore we also tried to verify the expression of the unprocessed LEFNAT in hematologic disease models. However, after several attempts and multiple optimizations of PCR conditions, we were not able to amplify the complete unspliced transcript using cDNA from 6 myeloid and 5 lymphoid cell lines. We also used the qRT-PCR primers used previously to measure LEFNAT in a subset of cDNA samples prepared with random primers. The amplification in myeloid cells and hela was in the limit of reliable detection of the equipment (Ct approx. 33 cycles), and amplification was around 5 cycles later than LEF1-AS1 in all samples, suggesting LEFNAT has very low expression in these cells at least under basal conditions.

PCR of NAT unspliced : The unprocessed transcript was only amplified in genomic DNA. We could not detect the transcript in cDNA from the following cell lines.

**Supplementary figure 3.**


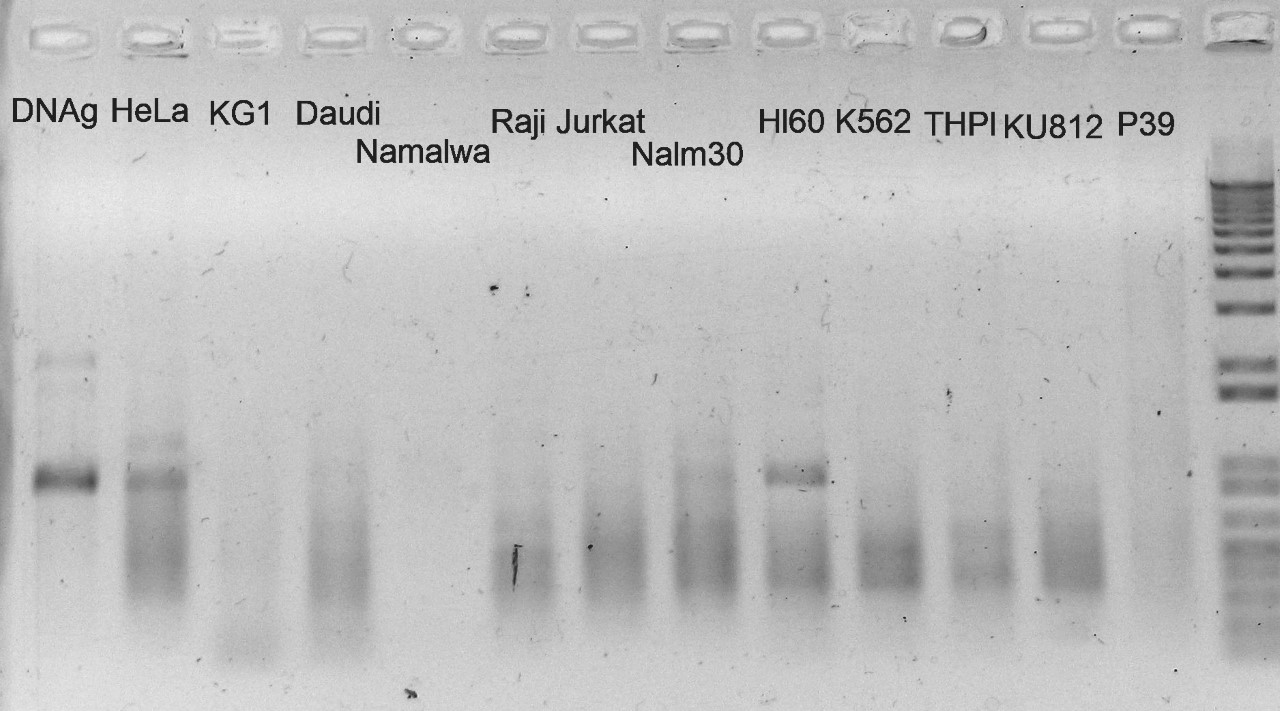


LEF1-NAT unspliced ~2Kb

**LEF1-AS1 and LEF1 expression level in association with patient characteristics**

In the patient (MDS and AML) group, LEF1 and LEF1-AS1 expression levels were not correlated with age ( LEF1-AS1/age: P value (two-tailed)=0.2868, Pearson r=-0.1726, 95% confidence interval : -0.4595 to 0.1468 ; LEF1/age: P value (two-tailed)=0.5814, Pearson r=-0.09106, 95% confidence interval: -0.3953 to 0.2312 ). Additionally, we also analyzed a potential correlation with percentage of blasts in the bone marrow, we found no significant correlation (LEF1/%blasts : P value= 0.1244, Pearson r= -0.2536, 95% confidence interval: -0.5304 to 0.07194; LEF1-AS1/%blasts: pvalue= 0.3715, Pearson r = -0.1471, 95% confidence interval: -0.4422 to 0.1767).

Interestingly, despite strong correlation between LEF1 and LEF1-AS1 expression (P value (two-tailed)< 0.0001, Pearson r = 0.7843, 95% confidence interval = 0.6537 to 0.8695), only LEF1-AS1 expression was positively correlated with AML patient survival (LEF1-AS1: P value (two-tailed)=0.0423 Pearson r=0.3934, 95% confidence interval=0.01567 to 0.6729).

**Supplementary figure 4.** Correlation and Linear regression between lef1 and LEF1-AS1 expression


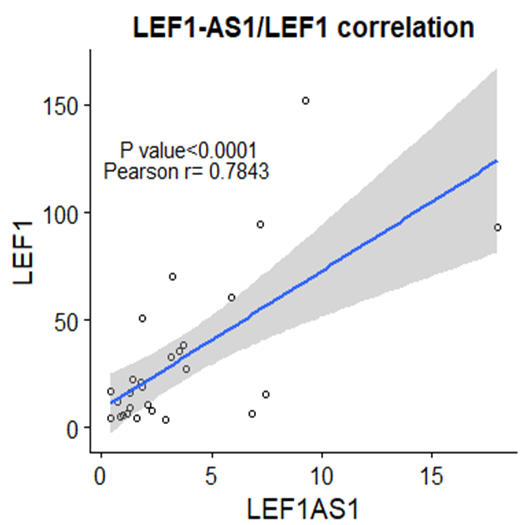


There was no significant correlation between LEF1 expression and time of survival of patients (P value (two-tailed)= 0.1962, Pearson r =0.2619, 95% confidence interval: -0.1397 to 0.5895).

**Supplementary figure 5.**

.
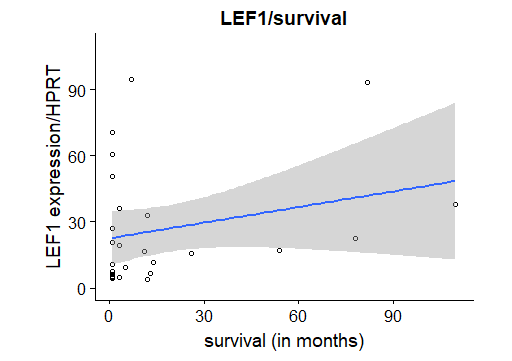


**LEF1-AS1 over-expression modulates important proliferation pathways**

In line with the observed reduction in proliferation, an increased expression of tumor suppressors CDKN1A (p21) and CDKN1B (p27) was detected in the mRNA and protein levels (supp. Fig. 6). A reduction of ERK1/2 activation was also detected by western blot, without modulation of ERK1/2 expression.

**Supplementary figure 6.** RT-PCR quantification of CDKN1A, CDKN1B and ERK1/2 and western blots.


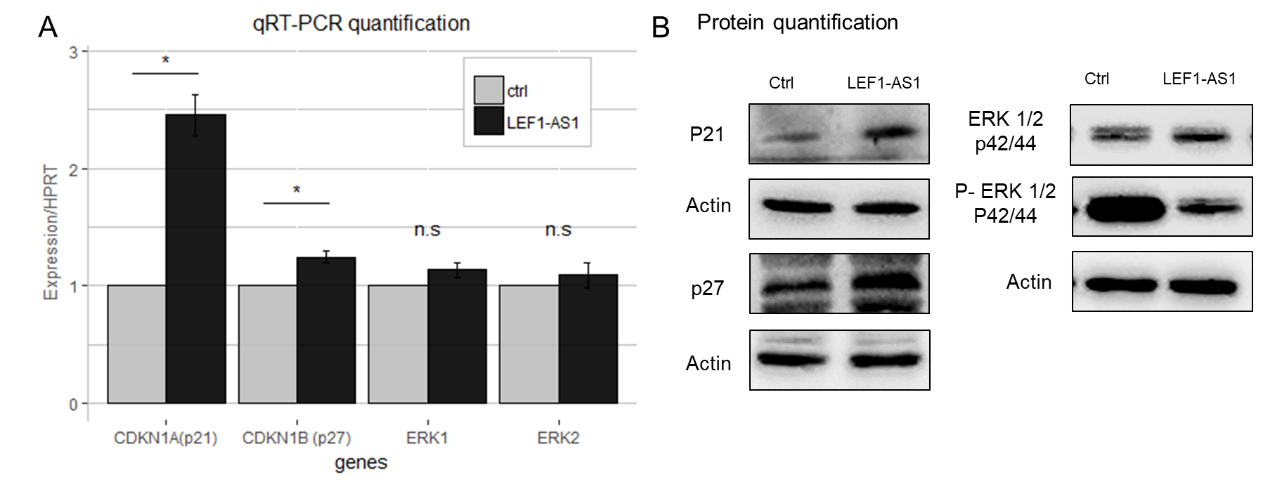


**Apoptosis – Cytometric analysis**

Annexin V staining showed no significant difference in apoptosis levels between control and LEF1-AS1 over-expressing cells, in both cell lines (HL60 and Hela). Cells were evaluated while growing exponentially.

**Supplementary figure 7.**


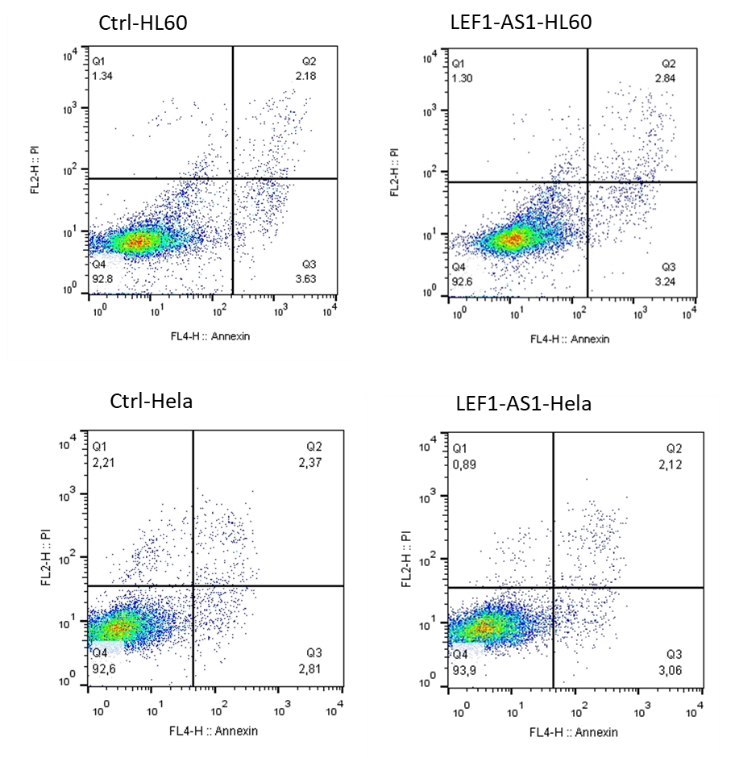


**Ki67 cytometric analysis in Hela**

Mean of fluorescence intensity of LEF1-AS1 over-expressing Hela cells was reduced compared to controls in cells growing exponentially (n=2). The cytometric histogram (left panel) shows a population of cells expressing lower levels of Ki67 in the LEF1-AS1 cells, indicating reduced proliferation. Control cells were all expressing high levels of ki67, indicating a higher proliferation rate.

**Supplementary figure 8.** Fluorescence intensity of Ki67 in hela control and LEF1-AS1 over-expressing cells (n=2)


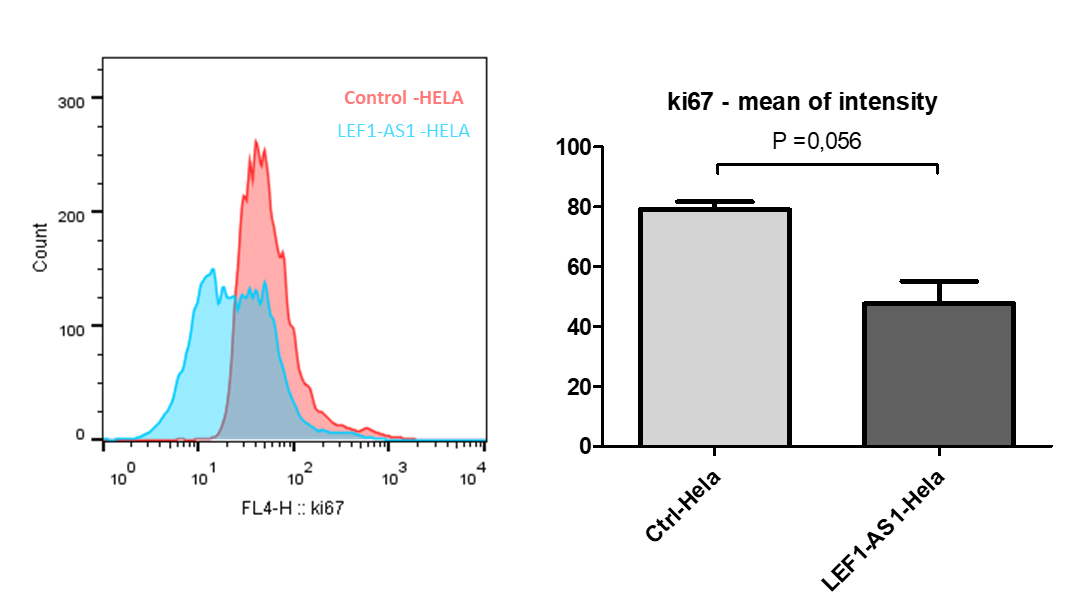


**MS-proteomics revealed a set of modulated proteins by LEF1-AS1 over-expression**

To characterize the function of LEF1-AS1 in cell physiology, we investigated differentially expressed proteins among the entire protein content of LEF1-AS1 and empty vector over-expressing cells. A total of over 500 proteins were identified (see complete list in a supplementary file) from this analysis and 16 were differentially expressed (P-value cut-off of 0.05, see table below).

**Supplementary table 4.** List of differentially expressed proteins from the Q-tof proteomic quantification (p-value cut-off 0.05)

| Protein | Gene name | Control (Mean, n=3) | Lef1-AS1 (Mean, n=3) | Pvalue |
| --- | --- | --- | --- | --- |
| Up-regulated proteins | | | | |
| **Talin-1*** | TLN1 | 60.92 | 95.87 | 0.0263 |
| 40S ribosomal protein S12 | RPS12 | 2.87 | 5.88 | 0.03594 |
| Actin-related protein 2/3 complex subunit 4 | ARPC4-TTLL3 | 2.22 | 4.55 | 0.01086 |
| Leukosialin | SPN | 1.91 | 2.9 | 0.04991 |
| Acidic leucine-rich nuclear phosphoprotein 32 family member B | ANP32B | 2.79 | 5.62 | 0.035249 |
| **Ras-related protein Rab-7a*** | RAB7A | 0 | 3.17 | 0.000186 |
| Apoptotic chromatin condensation inducer in the nucleus | ACIN1 | 0.43 | 2.7 | 0.035477 |
| Down-regulated proteins | | | | |
| 40S ribosomal protein S17 | RPS17 | 9.55 | 8.41 | 0.000205 |
| **Histone H3.1*** | HIST1H3A | 7.94 | 3.62 | 0.03478 |
| Elongation factor Tu, mitochondrial | TUFM | 8.8 | 7.52 | 0.008995 |
| Exportin-2 | CSE1L | 5.57 | 4.1 | 0.012933 |
| F-actin-capping protein subunit alpha-1 | CAPZA1 | 6.11 | 3.1 | 0.002007 |
| Dolichyl-diphosphooligosaccharide-protein glycosyltransferase subunit 2 | RPN2 | 10.29 | 3.13 | 0.02369 |
| Isoform of P55769, NHP2-like protein 1 | SNU13 | 2.54 | 0.98 | 0.00705 |
| **Fumarate hydratase, mitochondrial *** | FH | 1.7 | 0 | 0.010594 |
| Isoform of O75340, HCG1985580 | PDCD6 | 1.91 | 0 | 0.02502 |

**LEF1-AS1 is an exosome-contained circulating transcript**

Functional and cellular component enrichment analysis from the proteomic screening revealed that most of the differentially expressed proteins are associated with the exosomes. In addition, RAB7A is involved in exosome cargo selection and has been associated with the selective export of a regulatory non-coding RNA through exosomes[22]. These findings led us to hypothesize that LEF1-AS1 is being packed into exosomes, and that the observed increase in RAB7A and other exosome-associated proteins could be due to their potential role in the vesicular secretion of LEF1-AS1. We decided to investigate if LEF1-AS1 is secreted into the extracellular space and possibly released into the blood stream.

Exosomes are extracellular vesicles that are secreted by one cell, circulate in body fluids and taken up by other cells. Recent evidence revealed that they are critical in cell-to-cell communication and mediate several pathological conditions[15, 24, 26].

To investigate if LEF1-AS1 is packed and released in exosomes, we separated exosomes from blood using a commercial membrane affinity method (Qiagen Exoeasy columns) previously validated for efficiency and purity [40], that allows exosomes isolation and RNA extraction from serum. For exosomes extraction from culture medium we used a commercial proprietary polymer (ExoQuick-TC) also validated for exosome purity elsewhere[42]. We detected LEF1-AS1 transcript in exosomes isolated from the culture medium of HL60 and exosomes from blood serum of 3 healthy volunteers, but not in exosome-depleted serum (supplementary fig. 10).

Functional and cellular component enrichment analysis from the proteomic screening revealed that most of the differentially expressed proteins are associated with the exosomes (sup. Figure 9). Exosomes are extracellular vesicles that are secreted by one cell, circulate in body fluids and taken up by other cells. Recent evidence revealed that they are critical in cell-to-cell communication and mediate several pathological conditions[15, 24, 26].

**Supplementary figure 9.** Gene ontology enrichment of proteomics results, carried out using String software V10.5, FDR (false discovery rate) P values were generated by String software and plotted using “R.

**
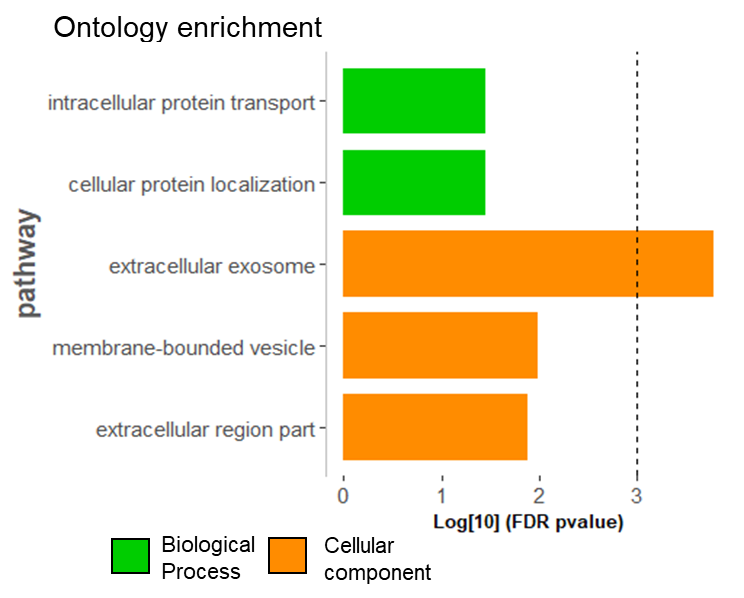
**

**Supplementary figure 10.**


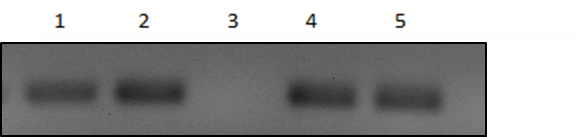


1: Serum-derived exosomes from donor 1

2: HL60- derived exosomes obtained from growth medium

3: Negative control 🡪 exosome-depleted serum amplification

4: Serum-derived exosomes from donor 2

5: Serum-derived exosomes from donor 3

Primers used : Fw GCATTGGGCAAGCACGTAA , Rv ACCTTGTTCTGCATTAATCTCCCTAT

**CFSE and Ki67 flow cytometry analysis and statistical results of 3 independent experiments**

Carboxyfluorescein succinimidyl ester (CFSE) labelling was used to trace cell divisions in HL60 stably transfected cells. Cell lines were stained with CFSE and cultured for 24h. Flow cytometry analysis showed that cells over-expressing LEF1-AS1 underwent less cell divisions (supp. Fig 11.A & B).

Flow cytometry measurement of Ki67 staining was also used as proliferation specific marker in synchronized cultures. The fluorescence intensity of the Ki67 antigen in the LEF1-AS1-HL60 cells was significantly reduced (supplementary figure 11B&C).

**Supplementary figure 11**. (A)CFSE cell division tracking assay of empty vector and LEF1-AS1-Hela. CFSE fluorescence was measured by flow cytometry 24h after labelling. Blue peak: parent cells, orange peak: first generation, green peak: second generation, purple peak: third generation. (B) Bar chart showing proliferation indexes calculated based on the number of cell divisions by Modfit software (n=3, mean ± SEM ). (C) Microscopic images of control and LEF1-AS1 Hela cells before cytometric measurements.


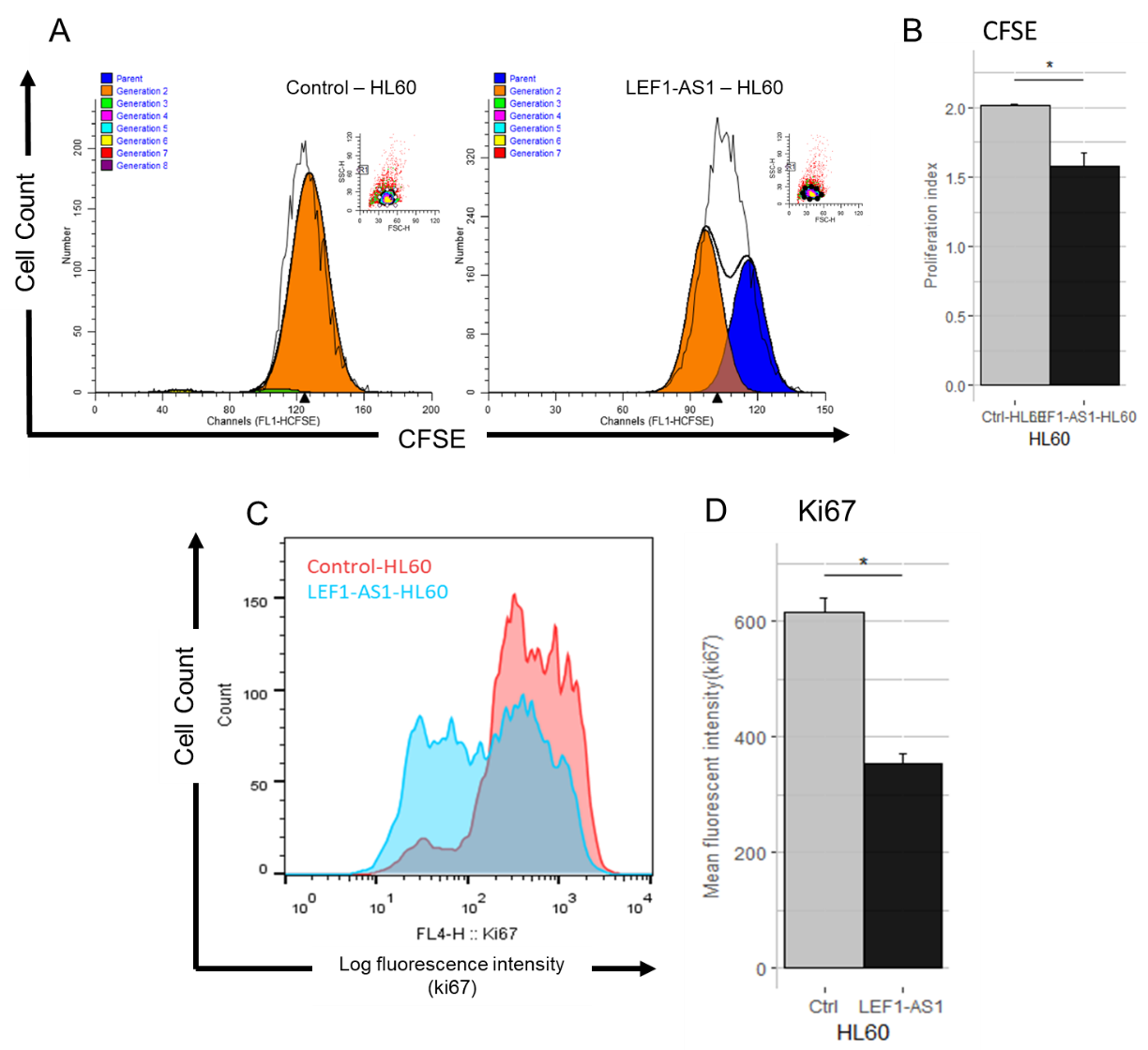


Supplementary figure 11

**Western blots**

**HIF1A**

Accumulation of HIF1A has been reported in fumarase-deficient tumors; we could not detect significant difference of HIF1A protein levels in our samples. However, HIF1A accumulation was also not observed in fumarase-deficient HSCs [20], and possibly the signaling downstream of fumarase in the hematopoietic system is not mediated by HIF1A.

**Supplementary figures 12-19**. Western blots of independent experiments

Unprocessed Hif1 alpha should appear at ~90Kda and processed protein at ~110 -120 Kda.


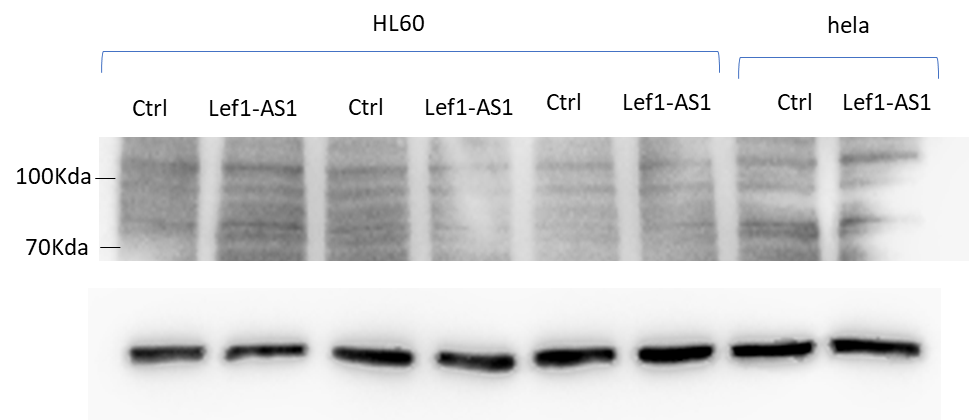


**LEF1: gel 10%**


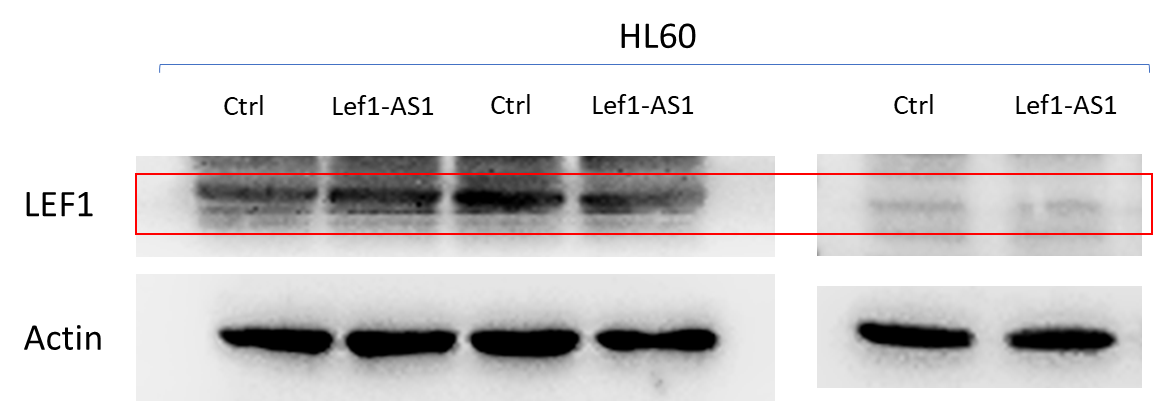


**P27: gel 10%**


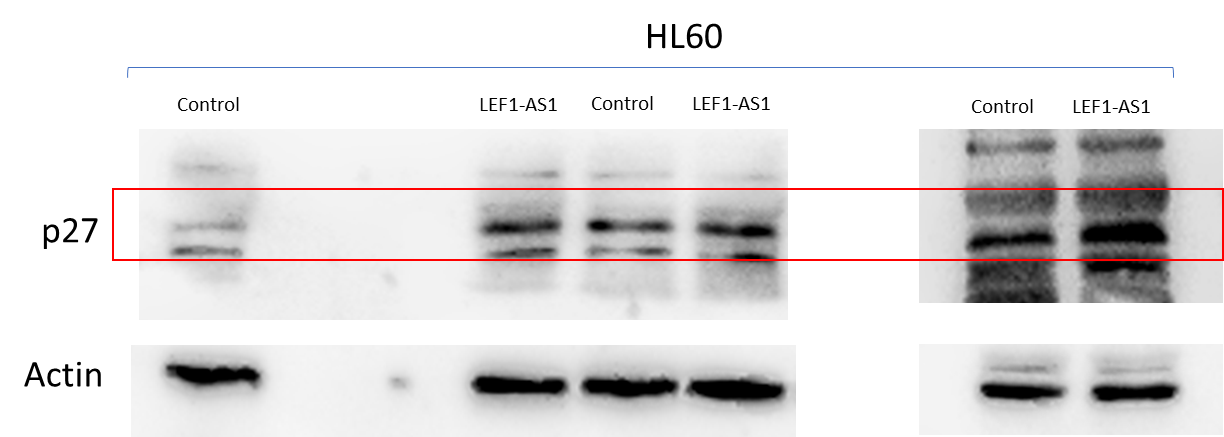


**P21: gel 10%**


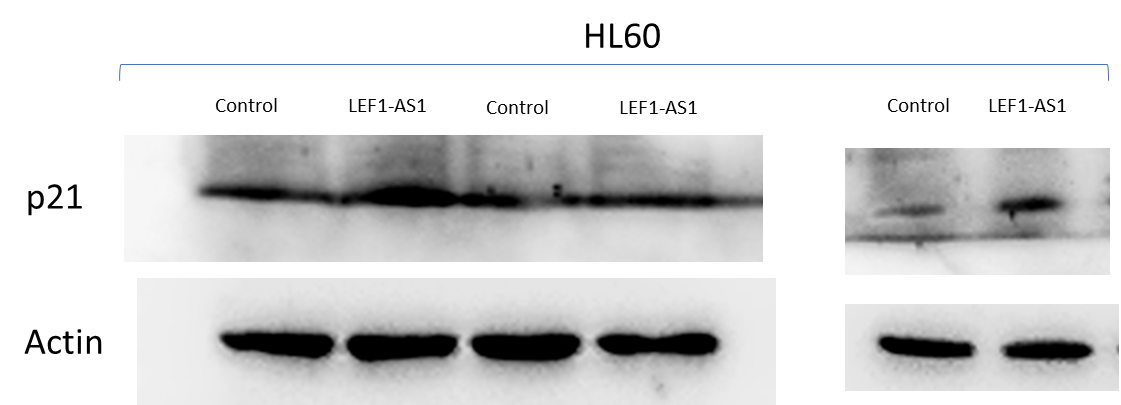


Talin 1: gel 8%


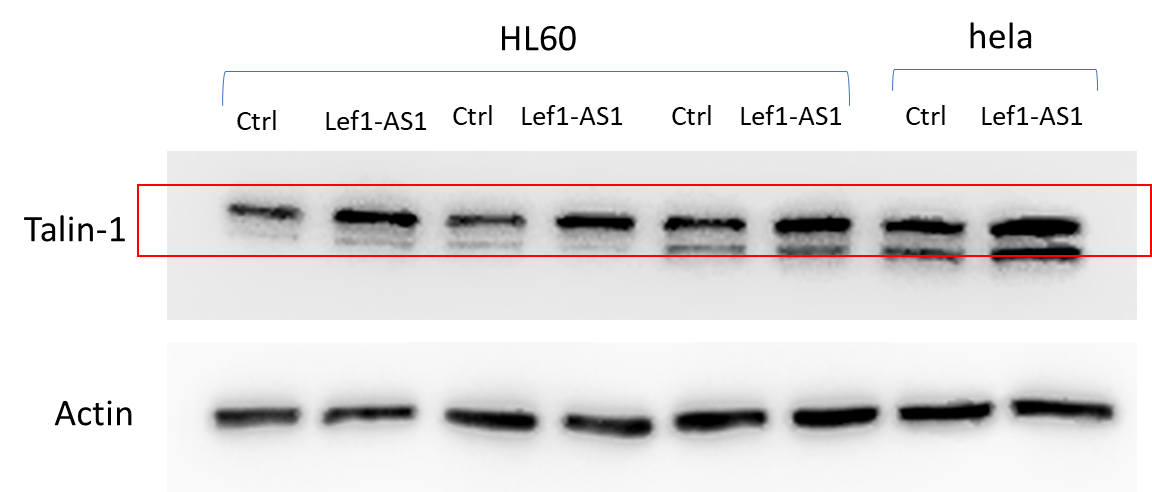


Histone 3: gel 15%


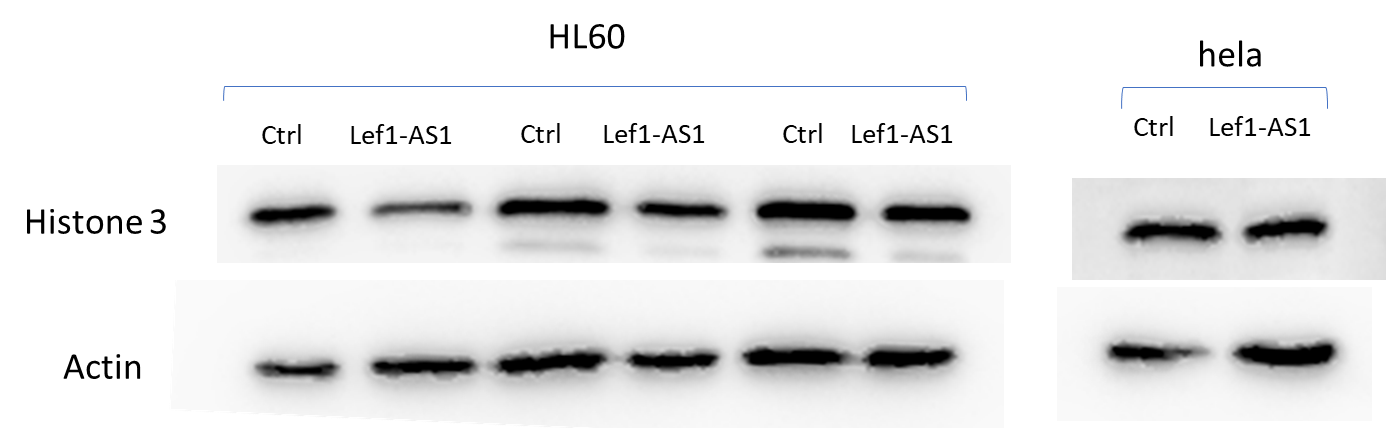


Fumarase: gel 10%


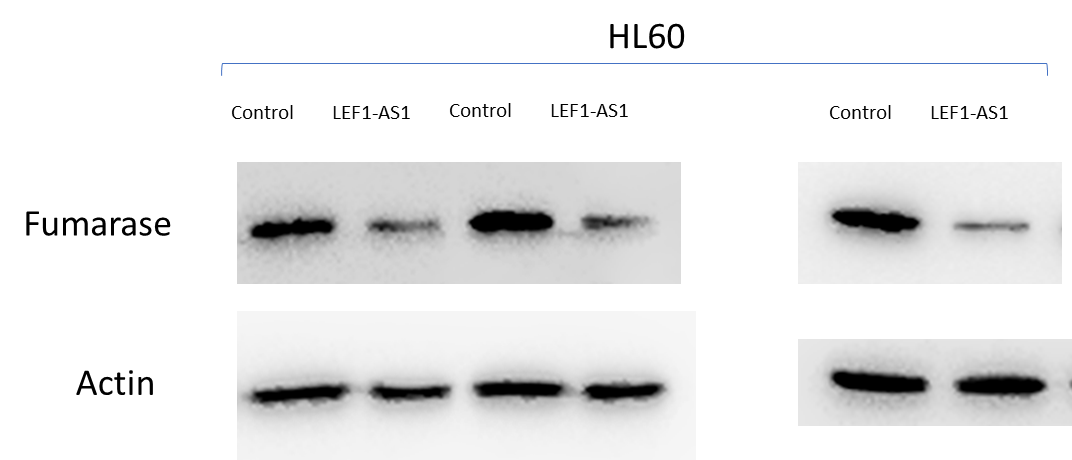


RAB7A : gel 12%


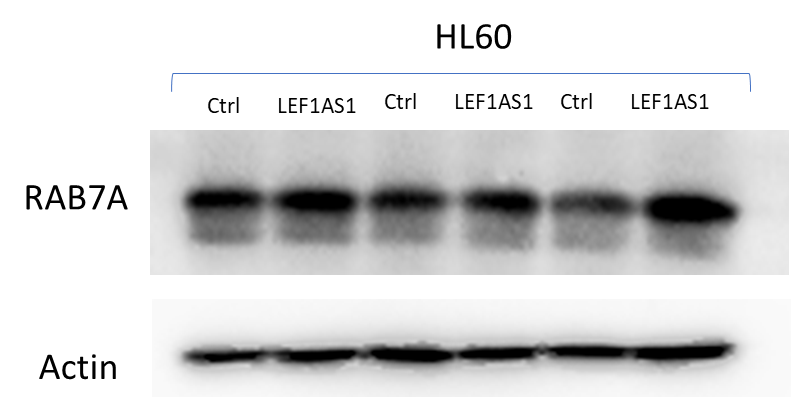

Supplement: Supplementary file 1 [file JCMM-23-3021-s001.docx]
